# Rivaroxabana em Pacientes Ambulatoriais com COVID-19 Leve ou Moderada: Fundamentação e Desenho do Estudo CARE (CARE – Coalition COVID-19 Brazil VIII)

**DOI:** 10.36660/abc.20220431

**Published:** 2023-03-20

**Authors:** Gustavo B. F. Oliveira, Precil Diego M. M. Neves, Haliton A. Oliveira, Daniela Ghidetti Mangas Catarino, Lucas B. O. Alves, Alexandre B. Cavalcanti, Regis G. Rosa, Viviane C. Veiga, Luciano C.P. Azevedo, Otávio Berwanger, Renato D. Lopes, Álvaro Avezum

**Affiliations:** 1 Centro Internacional de Pesquisa Hospital Alemão Oswaldo Cruz São Paulo SP Brasil Centro Internacional de Pesquisa, Hospital Alemão Oswaldo Cruz, São Paulo, SP – Brasil; 2 Instituto de Pesquisas HCOR São Paulo SP Brasil Instituto de Pesquisas HCOR, São Paulo, SP – Brasil; 3 Hospital Moinhos de Vento Porto Alegre RS Brasil Hospital Moinhos de Vento, Porto Alegre, RS – Brasil; 4 A Beneficência Portuguesa de São Paulo São Paulo SP Brasil A Beneficência Portuguesa de São Paulo, São Paulo, SP – Brasil; 5 Hospital Sírio Libanês Instituto de Pesquisa e Ensino São Paulo SP Brasil Hospital Sírio Libanês – Instituto de Pesquisa e Ensino, São Paulo, SP – Brasil; 6 Hospital Israelita Albert Einstein São Paulo SP Brasil Hospital Israelita Albert Einstein, São Paulo, SP – Brasil; 7 Duke University Medical Center Durham NC EUA Duke University Medical Center, Durham, NC – EUA; 8 Instituto Brasileiro de Pesquisas Clínicas São Paulo SP Brasil Instituto Brasileiro de Pesquisas Clínicas, São Paulo, SP – Brasil

**Keywords:** COVID-19, Trombose, Resultado do Tratamento, Rivaroxabana, Ambulatório Hospitalar

## Abstract

**Fundamento:**

Estudos anteriores revelaram alto risco de eventos tromboembólicos arteriais e venosos como consequência de danos virais diretos do SARS-CoV-2 em células endoteliais e um meio procoagulante devido ao aumento de biomarcadores como o D-dímero, fibrinogênio, fator VIII. Foram realizados ensaios controlados randomizados de terapias antitrombóticas em pacientes internados, no entanto, poucos estudos avaliaram o papel da tromboprofilaxia no ambiente ambulatorial.

**Objetivo:**

Avaliar se a profilaxia antitrombótica com rivaroxabana reduz o risco de eventos trombóticos venosos ou arteriais, suporte ventilatório invasivo e morte em pacientes ambulatoriais com COVID-19.

**Métodos:**

O estudo CARE é um ensaio randomizado, aberto, multicêntrico e controlado por rivaroxabana 10 mg uma vez por dia durante 14 dias ou tratamento local padrão isolado, para a prevenção de resultados adversos, registrado no Clinicaltrials.gov (NCT04757857). Os critérios de inclusão são adultos com infecção confirmada ou suspeita do SARS-CoV-2, com sintomas leves ou moderados, sem indicação de hospitalização, no prazo de 7 dias após o início dos sintomas e um fator de risco de complicação da COVID-19 (>65 anos, hipertensão, diabetes, asma, doença pulmonar obstrutiva crônica ou outras doenças pulmonares crônicas, tabagismo, imunossupressão ou obesidade). O desfecho primário composto inclui tromboembolismo venoso, necessidade de ventilação mecânica invasiva, eventos cardiovasculares agudos maiores e mortalidade no prazo de 30 dias após a randomização, sendo avaliado segundo o princípio da intenção de tratar. Todos os pacientes assinaram termo de consentimento. Foi estabelecido um nível de significância de 5% para todos os testes estatísticos.

**Resultados:**

Os principais desfechos trombóticos e hemorrágicos, hospitalizações e mortes serão avaliados centralmente por um comitê de eventos clínicos independente, sob a condição cega para a alocação dos grupos de tratamento.

**Conclusão:**

O estudo CARE fornecerá informação relevante e contemporânea sobre o possível papel da tromboprofilaxia em pacientes ambulatoriais com COVID-19.

Clinical trials registration: NCT04757857

## Introdução

Os sintomas mais comuns da doença do Coronavírus 2019 (COVID-19) incluem febre, fadiga, tosse seca, anorexia, mialgia e dispneia.^1 - 7^ No entanto, os pacientes podem progredir para a síndrome respiratória aguda grave (SARS) e síndrome inflamatória multissistêmica com elevado risco de morte.^3 - 6 , 8 - 15^ Além disso, foi relatado elevado risco de eventos trombóticos arteriais e venosos como consequência de danos virais diretos de células endoteliais e aumento de fatores procoagulantes tais como D-dímero, fibrinogênio, fator VIII, bem como estado de hiperviscosidade,^16 - 18^ o que intensifica a trombose micro e macrovascular^19 , 20^ com uma relação direta com a gravidade da COVID-19.^21 - 25^ Além disso, vários estudos realizados no contexto epidemiológico e sanitário brasileiro relataram uma série de fatores de risco cardiovascular associados tanto à sobrecarga como ao prognóstico da COVID-19.^26 - 29^ Foram realizados ensaios controlados randomizados de terapias antitrombóticas em pacientes com COVID-19 hospitalizados, especialmente em pacientes críticos em unidades de terapia intensiva.^30 - 35^ De fato, as diretrizes atuais recomendam a profilaxia antitrombótica com heparina de baixo peso molecular (LMWH) ou heparina não fracionada (UFH) para pacientes hospitalizados.^36 - 40^ Contudo, apesar dos relatos de eventos trombóticos ambulatoriais,^41^ foram publicados poucos estudos sobre a sua frequência e o possível papel da tromboprofilaxia.^30^ A rivaroxabana é um inibidor direto e altamente seletivo do fator Xa, com eficácia comprovada para a tromboprofilaxia^42 , 43^ e para a prevenção cardiovascular secundária.^44^ Os inibidores seletivos do FXa podem deter a ampliação de geração de trombina.^45^

O objetivo deste estudo é avaliar se a rivaroxabana reduz os desfechos compostos de eventos do tromboembolismo venoso (TEV), necessidade de ventilação mecânica invasiva, eventos cardiovasculares adversos maiores (ECAM), definido como infarto agudo do miocárdio, acidente vascular cerebral ou isquemia aguda de membros e morte dentro ou fora do hospital no prazo de 30 dias após a randomização de pacientes ambulatoriais com COVID-19, sem indicação clara de hospitalização após assistência médica inicial.

## Métodos

### Coalizão COVID-19 Brasil

A Coalizão COVID-19 Brasil é uma iniciativa de investigação colaborativa multicêntrica de pesquisadores brasileiros com o objetivo de avaliar os benefícios de diferentes fármacos para diferentes níveis de gravidade da Covid-19. Alguns estudos foram publicados desde então como resultado dessa rede, os quais avaliaram efeitos de tratamentos como azitromicina, hidroxicloroquina, dexametasona, tocilizumabe e rivaroxabana em pacientes moderados a graves.^46 - 51^

### Desenho do estudo

Trata-se de ensaio controlado randomizado (1:1), aberto e multicêntrico de rivaroxabana (10 mg VO uma vez por dia durante 14 dias) em comparação com o tratamento local padrão, dentro de 7 dias a partir do início dos sintomas, para reduzir os desfechos compostos de TEV, a necessidade de ventilação mecânica invasiva, ECAM e a mortalidade devido à COVID-19 dentro de 30 dias a partir da randomização. O estudo foi realizado em 47 locais com base em avaliação de viabilidade favorável, cumprimento de boas práticas clínicas e aprovação ética. O protocolo segue as declarações do *Standard Protocol Items: Recommendations for Interventional Trials* .^52^ A Ilustração Central exibe o fluxograma do estudo.

**Figura Central:**
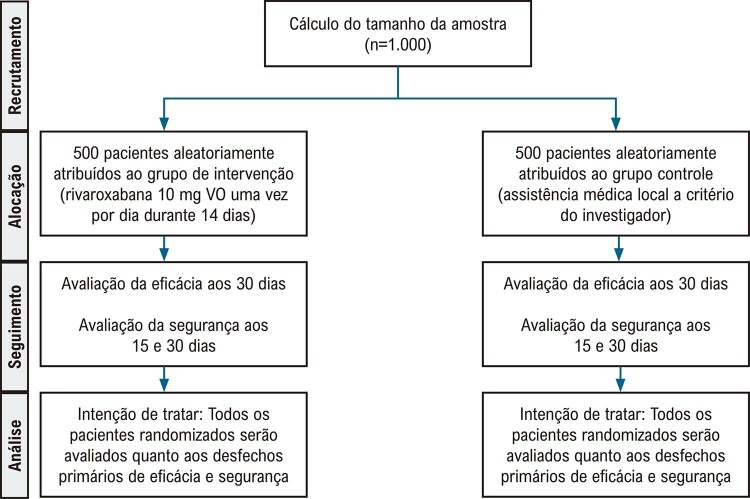
: Rivaroxabana em Pacientes Ambulatoriais com COVID-19 Leve ou Moderada: Fundamentação e Desenho do Estudo CARE (CARE – Coalition COVID-19 Brazil VIII)

*Diagrama CONSORT mostrando o fluxograma do estudo e o recrutamento planejado.*

### Desfechos primários e secundários

Avaliaremos o desfecho de eficácia composto por TEV, necessidade de ventilação mecânica invasiva, ECAM e morte dentro ou fora do hospital após 30 dias de seguimento. Também pretendemos avaliar o tempo da randomização à hospitalização, a duração da frequência de admissão à unidade de terapia intensiva, necessidade de cuidado e a duração da ventilação mecânica, o desfecho vascular composto I, (definido como infarto do miocárdio não fatal, acidente vascular cerebral isquêmico não fatal ou morte cardiovascular, TEV), o desfecho vascular composto II, (definido como morte cardiovascular, infarto do miocárdio não fatal, acidente vascular cerebral isquêmico não fatal ou isquemia aguda de membros, TEV), hemorragia importante definida pelos critérios da Sociedade Internacional de Trombose e Hemostasia (ISTH):^53^ a) Qualquer sinal ou sintoma de hemorragia (hemorragia que exceda o esperado para uma circunstância clínica, incluindo hemorragia diagnosticada apenas por imagem) que não se enquadre nos critérios da hemorragia maior da ISTH, mas que satisfaça pelo menos um dos critérios seguintes: i. Que requeira intervenção médica por profissional de saúde; ii. Que conduza à hospitalização ou ao aumento do nível de assistência; iii. Que provoque uma avaliação presencial (não apenas por telefone ou comunicação eletrônica); b) A hemorragia grave definida pela ISTH em pacientes não cirúrgicos é definida como tendo uma apresentação sintomática e: i. Hemorragia fatal, e/ou ii. Hemorragia numa área ou órgão crítico, como intracraniana, intraespinhal, intraocular, retroperitoneal, intra-articular ou pericárdica ou intramuscular com síndrome compartimental e/ou iii. Hemorragia que provoque diminuição do nível de hemoglobina de 20 g.L^-1^ (1,24 mmol.L^-1^) ou mais ou que conduza à transfusão de duas ou mais unidades de sangue total ou eritrócitos e mortalidade por todas as causas.

### Critérios de seleção

Casos suspeitos e confirmados serão definidos com base na classificação definida nas Diretrizes do Ministério da Saúde do Brasil e recomendações da OMS^54 , 55^ e adaptada para o ambiente ambulatorial ( Tabela 1 ). Os critérios de inclusão e exclusão são descritos na Tabela 2 .

**Tabela 1:** – Definição de caso de COVID-19 para o Estudo CARE conforme o Ministério da Saúde do Brasil e a Organização Mundial da Saúde

Estado da COVID-19	Definição
Confirmado	Indivíduo com confirmação laboratorial de COVID-19 (detecção do vírus SARS-CoV-2 por meio de RT-PCR), de preferência coletado entre o 4º e 7º dia de início dos sintomas por esfregaços nasofaríngeos/orofaríngeos, independentemente dos sinais e sintomas. Ensaio imunocromatográfico rápido validado para detecção de antígeno viral em amostra coletada das vias respiratórias superiores, de preferência entre o 2º e o 7º dia após o início dos sinais e sintomas. Imunológicos (teste rápido ou sorologia clássica para detectar anticorpos IgM/IgG), numa amostra coletada após o 7º dia de início dos sintomas, analisados por teste validado.
Suspeito	Paciente que preencha pelo menos um dos seguintes critérios *: Paciente com doença respiratória aguda (febre E pelo menos um sinal/sintoma de doença respiratória, por exemplo, tosse ou dispneia) E histórico de viagem ou residente em local que relata a transmissão comunitária da COVID-19 durante os 14 dias anteriores ao início dos sintomas. Paciente com doença respiratória aguda E tendo estado em contato com um caso confirmado ou provável (com doença respiratória aguda sem confirmação laboratorial) de COVID-19 nos 14 dias anteriores ao início dos sintomas.

* Dependendo do estado clínico do paciente, esses critérios podem ser complementados por achados radiológicos (infiltração intersticial na radiografia do tórax e/ou opacidade em vidro fosco na tomografia pulmonar). Deve salientar-se que trataremos uma maioria com sintomas leves, que não têm indicação clínica para exame por imagem.

**Tabela 2:** – Critérios de inclusão e exclusão

Critérios Gerais
Adultos ambulatoriais (>18 anos) à procura de assistência médica com diagnóstico suspeito ou confirmado de COVID-19, com ≤ 7 dias desde o início dos sintomas, apresentando sintomas leves a moderados sem indicação clara de hospitalização e pelo menos dois dos seguintes fatores de risco de complicações:

**Critérios de inclusão**

≥65 anos Hipertensão arterial Diabetes Asma DPOC ou outras doenças pulmonares crônicas Tabagismo Imunossupressão Obesidade (IMC > 30) Histórico de câncer não ativo Restrição de leito ou mobilidade reduzida (≥50% do tempo de vigília sem deambular) Histórico anterior de TEV Uso de contraceptivos hormonais orais

**Critérios de Exclusão**

Pacientes <18 anos de idade; Indicação de hospitalização na primeira assistência médica; Teste positivo para influenza na primeira consulta; Qualquer doença hepática conhecida associada à coagulopatia; RNI> 1,5; Gestantes, lactantes ou com a possibilidade de engravidar e sem utilizar um método contraceptivo adequado; Alto risco de hemorragia; histórico de bronquiectasia ou cavitação pulmonar, hemorragia significativa nos últimos 3 meses, úlcera gastroduodenal ativa, histórico de hemorragia recente (dentro de 3 meses) ou elevado risco de hemorragia; Acidente vascular cerebral no prazo de 1 mês ou qualquer histórico de acidente vascular cerebral hemorrágico ou lacunar ou qualquer hemorragia intracraniana ou qualquer neoplasia intracraniana, metástase cerebral, malformação arteriovenosa ou aneurisma cerebral; Insuficiência cardíaca grave com fração de ejeção do ventrículo esquerdo <30% (identificada por ecocardiograma ou outro método validado previamente documentado) ou sintomas de insuficiência cardíaca classe III ou IV da New York Heart Association (NYHA); Taxa de filtração glomerular estimada (TFGe) <30 mL/min conforme a equação CKD-EPI; Indicação clínica para terapia antiplaquetária dupla ou terapia anticoagulante (TEV, fibrilação atrial/ *flutter* , prótese de válvula mecânica); Trombocitopenia marcada (plaquetas <50.000/mm^3^); Doenças não cardiovasculares associadas a prognóstico desfavorável, por exemplo, neoplasias ativas (excluindo câncer de pele não melanoma) definidas como neoplasias sem remissão ou que requerem quimioterapia ativa ou terapias adjuvantes como imunoterapia ou radioterapia, ou que aumentam o risco de reação adversa às intervenções avaliadas; Histórico de hipersensibilidade ou contraindicação conhecida à rivaroxabana; Tratamento sistêmico com fortes inibidores do CYP3A4 e glicoproteína p (P-gp) (por exemplo, antimicóticos azólicos sistêmicos, tais como cetoconazol e inibidores da proteína do vírus da imunodeficiência humana [HIV], tais como ritonavir) ou fortes indutores do CYP3A4, ou seja, rifampicina, rifabutina, fenobarbital, fenitoína e carbamazepina; Tratamento existente em teste; Participação concomitante em outro estudo com drogas experimentais no contexto da COVID-19; Uso de cloroquina ou hidroxicloroquina associado à azitromicina; Câncer ativo; Outras contraindicações à rivaroxabana;

*RNI: razão normalizada internacional; CKD-EPI: Chronic kidney disease epidemiology collaboration.*

### Intervenções e avaliação de seguimento

Ambos os braços se destinam a receber assistência padronizada conforme a prática local, que inclui recomendações gerais e medicamentos para alívio dos sintomas que ficam a critério médico. Os pacientes do grupo rivaroxabana recebem 10 mg VO uma vez por dia durante 14 dias. Os pacientes do grupo controle não devem receber tratamento complementar para além da assistência local, como mencionado anteriormente. Estão previstas duas consultas de seguimento através de chamadas telefônicas para avaliar a adesão à rivaroxabana e detectar possível progressão da doença ou eventos adversos.

### Interrupção do medicamento e seguimento do paciente

O investigador interromperá o medicamento em estudo e o paciente deverá ser seguido por 30 dias e todos os procedimentos delineados neste protocolo deverão ser realizados nesses casos:

- Qualquer condição médica que, a critério do patrocinador ou do investigador, impeça o paciente de continuar o tratamento, descrevendo o motivo e a respectiva evidência;
- Os pacientes desenvolverem um evento adverso grave que compromete a adesão e a segurança. O investigador seguirá os pacientes até que o evento adverso esteja resolvido ou estável e seja considerado clinicamente irrelevante.
- Pacientes com resultado negativo para SARS-CoV-2 pela reação da transcriptase reversa (RT-PCR).

Quando o teste para SARS-Cov-2 for realizado no hospital em que o paciente é randomizado, o investigador principal do centro acessará essa informação e a compartilhará com o centro coordenador (Centro Internacional de Pesquisa do Hospital Alemão Osvaldo Cruz). O paciente será informado da interrupção do tratamento e continuará sendo seguido até o fim do seguimento de 30 dias.

Quando o teste para SARS-Cov-2 (RT-PCR) for realizado em outro laboratório, o paciente informará o centro em que é randomizado e depois este informará o Centro Internacional de Pesquisa para que a equipe do estudo possa indicar a interrupção do medicamento mantendo o seguimento por até por até 30 dias.

O estudo será interrompido caso sejam observados benefícios claros da intervenção no desfecho primário de eficácia ou no caso de aumento da frequência de eventos hemorrágicos importantes. O Comitê de Monitoramento de Segurança de Dados ( *Data Safety Monitoring Board* , DSMB) avaliará atentamente qualquer ocorrência de eventos adversos graves e, através de pressupostos estatísticos, poderá recomendar o fim do estudo para garantir a segurança dos pacientes.

### Manejo e relato de eventos adversos

As informações coletadas devem incluir o histórico médico e as comorbidades, o diagnóstico clínico de COVID-19 e sua gravidade, a data de início, a definição da probabilidade de relação causal, bem como a causa principal, a decisão médica, os desfechos adversos, os critérios utilizados para classificar a gravidade do evento e a data do término.

### Relato e julgamento do desfecho

O desfecho primário de eficácia será avaliado por médicos com experiência prévia no julgamento de eventos clínicos. As admissões hospitalares devido a causas relacionadas com a COVID-19 serão documentadas pela equipe médica e a informação será coletada para verificação de eventos clínicos sob condição cega, seguindo critérios padronizados semelhantes aos já aplicados no estudo PURE (Prospective Urban and Rural Epidemiology).

### Coleta e tratamento de dados

**Figura 1:**
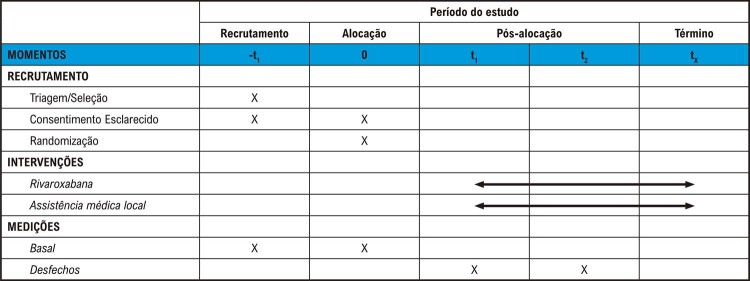
– Esquema de levantamento de dados e seguimento dos pacientes.

Os dados serão coletados por meio de formulário eletrônico de coleta de dados ( *electronic case report form, eCRF* ) e registados por cada centro participante, sendo a formação e o apoio fornecidos pelo centro coordenador para assegurar a qualidade dos dados. Os dados a serem coletados do paciente e/ou familiares durante as visitas de estudo incluem:

- 1. Admissão (início do estudo):

- Idade, sexo, estado civil, etnia, escolaridade, renda familiar e comorbidades;
- Resultados de testes moleculares ou sorológicos para COVID-19 (dependendo do tempo desde o início dos sintomas/diagnóstico clínico);
- Uso concomitante de medicamentos no início do estudo;
- Duração dos sintomas;

- 2. No 15º dia após a randomização:

- Avaliação da segurança (monitoramento de eventos adversos);
- Adesão ao medicamento;

- 3. No 30º dia após a randomização:

- Avaliação da eficácia (desfecho primário);
- Avaliação da segurança (monitoramento de eventos adversos);

A figura 1 apresenta o esquema da coleta de dados e do seguimento dos participantes.

### Análise estatística

#### Cálculo amostral e procedimento de randomização

Estimamos que uma amostra de 932 pacientes (466 por grupo) forneceria um poder estatístico de 80% para detectar uma redução do risco relativo (RRR) de 30%, pressupondo uma taxa de desfecho primário de 25% no Grupo Controle e 17,5% no Grupo Rivaroxabana, um teste de hipótese bidirecional e um nível de significância de 5% utilizando o teste Qui-quadrado. A taxa de perda de seguimento estimada foi de 5% por grupo, o que implicaria 982 pacientes. Finalmente, decidiu-se que 1.000 pacientes seriam randomizados no estudo. O cálculo do tamanho da amostra foi realizado com o SAS 9.4 (procedimento PROC POWER).^56 , 57^ A randomização será permutada em blocos com tamanho de bloco fixo de oito, por meio de uma lista de randomização gerada eletronicamente utilizando *software* próprio.^58 , 59^ A ocultação da alocação será mantida por um sistema 24 horas, centralizado, automatizado e via Internet desenvolvido pelo Hospital Alemão Oswaldo Cruz.

#### Análises interinas

Preveem-se três análises interinas por um DSMB independente quando o tamanho da amostra atingir 25% (250 participantes), 50% (500 participantes) e 75% (750 participantes) conforme a abordagem Haybittle-Peto.^60 , 61^ Na análise de segurança, o estudo pode ser interrompido conforme o método Haybittle-Peto se houver sinal de dano com p<0,01 (em cada análise interina). Na análise de eficácia, o estudo pode ser interrompido conforme o método de Haybittle-Peto se houver sinal de benefício (desfecho primário) com p<0,001 (em cada análise interina).^62^

#### Métodos estatísticos

As características basais serão referidas como frequência, porcentagem, média e desvio-padrão ou mediana e intervalo interquartil, sempre que apropriado.^63^ O efeito da rivaroxabana sobre os desfechos primários e secundários será estimado com risco relativo (RR) e intervalo de confiança de 95% (IC95%). Todos os desfechos serão avaliados por intenção de tratar. O teste Qui-quadrado ou o teste exato de Fisher serão aplicados para o teste de hipóteses. A diferença absoluta entre duas proporções e relatada com o respectivo IC95% será avaliada conforme o método de Newcombe.^64 , 65^ Análises exploratórias do desfecho primário serão feitas considerando o efeito da intervenção dentro de subgrupos predefinidos ou *post-hoc* , conforme observações ao longo do estudo e mudanças dinâmicas no surto de variantes do SARS-CoV-2. Realizar-se-ão testes de interação utilizando modelos de regressão logística binária que incluem o efeito do tratamento, o fator de interesse e um termo de interação entre as duas variáveis (termo de interação tratamento-subgrupo) utilizando o conjunto completo de pacientes e reportando o valor p do termo de interação.^66^ O efeito da intervenção sobre a mortalidade será avaliado utilizando curvas de Kaplan-Meier e modelo de regressão de risco proporcional univariado de Cox, com Hazard Ratio (HR) e IC95%. As curvas de sobrevida serão comparadas com testes *log-rank* . Os pressupostos de risco proporcional serão verificados usando somas cumulativas de resíduos de Martingale e teste supremo do tipo Kolmogorov com base numa amostra de 1.000 padrões residuais simulados.^67 , 68^ Se o fenômeno de probabilidade monótona ocorrer ou se for observado um número raro de eventos, a abordagem de probabilidade parcial penalizada de Firth no modelo de regressão de Cox será aplicada com HR e IC95% do perfil de probabilidade.^69 , 70^ Com base no recrutamento de pacientes e dados epidemiológicos continuamente atualizados pelo Ministério da Saúde e relatórios locais, pretendemos incluir três métodos adicionais que podem contribuir para a avaliação da eficácia clínica global: a) Desfecho primário de eficácia mais internações devido à COVID-19; b) Taxa de vitória ( *win-ratio* ) do desfecho primário de eficácia; e c) Taxa de vitória ( *win-ratio* ) do desfecho primário de eficácia mais internações devido à COVID-19.^71 , 72^ Os desfechos secundários definidos por variáveis quantitativas serão comparados entre os dois grupos utilizando o teste *t* de Student não pareado ou o teste Mann-Whitney para variáveis distribuídas não normalmente, quando apropriado. Proceder-se-á à comparação de eventos adversos entre os dois grupos de tratamento com o teste Qui-quadrado ou o teste exato de Fisher. Além disso, para o desfecho primário, se a proporção de dados em falta for superior a 5%,^73 , 74^ será realizada uma análise de sensibilidade utilizando uma técnica de imputação múltipla de dados.^75 , 76^ A normalidade será avaliada com inspeção visual dos histogramas e aplicação de testes de normalidade como o teste Shapiro-Wilk ou o teste D’Agostino-Pearson, quando conveniente.^77 , 78^ Todos os testes de hipóteses serão bidirecionais e um valor de p menor que 0,05 será considerado estatisticamente significativo. As análises estatísticas serão realizadas com o SAS versão 9.4 (SAS Institute Inc, Cary, NC, EUA) ou R Statistical Software (R Foundation, Viena, Áustria).

#### Bloqueio do banco de dados

O bloqueio do banco de dados ocorrerá após a conclusão do seguimento de 30 dias de todos os pacientes, monitoramento clínico final e monitoria dos dados. Todas as análises interinas serão postas à disposição das agências reguladoras locais do Brasil. O acesso ao banco de dados será concedido apenas aos membros do Comitê Diretor e aos estatísticos antes da publicação dos principais resultados.

#### Supervisão do ensaio clínico

O Comitê Executivo/Diretor é responsável pela supervisão geral do estudo, desenvolvimento do protocolo do estudo e redação do manuscrito. O DSMB avaliou os efeitos da rivaroxabana em comparação com os cuidados padronizados conforme a prática local em relação aos desfechos primários e secundários. Essa comissão supervisiona qualquer evento adverso grave e pode recomendar a interrupção do tratamento, se necessário, para garantir a segurança do paciente.

## Ética e divulgação

Os registos de todos os pacientes serão mantidos confidenciais e acessíveis de forma restrita. Cada paciente ou representante legal assinou termo de consentimento livre e esclarecido depois de os riscos/benefícios e procedimentos de estudo terem sido totalmente explicados. O protocolo de estudo inicial e todas as emendas foram aprovados pelos conselhos de revisão institucionais locais e pela Comissão Nacional de Ética e Pesquisa do Brasil, em conformidade com a Resolução CNS 466/2012. Este estudo está registado sob o número NCT04757857 no Clinicaltrials.gov. Este estudo será submetido para publicação independentemente dos seus resultados.

## Perspectiva

O estudo CARE fornecerá informação relevante e contemporânea sobre o possível contributo da tromboprofilaxia em pacientes ambulatoriais com COVID-19.

## Fontes de financiamento

Este estudo foi iniciado por investigadores com o apoio financeiro da COALIZÃO COVID-19 Brasil e Bayer S.A., que forneceram o medicamento em estudo e auxílio financeiro parcial. As fontes de financiamento não tiveram qualquer papel na realização e análise do estudo, interpretação dos dados ou decisão de publicar os resultados.

## Declaração de compartilhamento de dados

Todas as informações relativas a este protocolo de ensaio clínico estão acessíveis aqui.
