# Supplementary material for: Rivaroxabana em Pacientes Ambulatoriais com COVID-19 Leve ou Moderada: Fundamentação e Desenho do Estudo CARE (CARE – Coalition COVID-19 Brazil VIII)
Source: Arq Bras Cardiol. 2023 Mar 20;120(3):e20220431. [Article in Portuguese] doi: 10.36660/abc.20220431 (PMC10392853; doi:10.36660/abc.20220431)
Supplement: Translation

# Translation: Rivaroxaban in Outpatients with Mild or Moderate COVID-19: Rationale and Design of the Study CARE (CARE – Coalition COVID-19 Brazil VIII)

**Authors:** Gustavo B. F. Oliveira, Precil Diego M. M. Neves, Haliton A. Oliveira, Daniela Ghidetti Mangas Catarino, Lucas B. O. Alves, Alexandre B. Cavalcanti, Regis G. Rosa, Viviane C. Veiga, Luciano C.P. Azevedo, Otávio Berwanger, Renato D. Lopes, Álvaro Avezum

**Affiliations:** 1 Brazil Centro Internacional de Pesquisa, Hospital Alemão Oswaldo Cruz, São Paulo, SP – Brazil; 2 Brazil Instituto de Pesquisas HCOR, São Paulo, SP – Brazil; 3 Brazil Hospital Moinhos de Vento, Porto Alegre, RS – Brazil; 4 Brazil A Beneficência Portuguesa de São Paulo, São Paulo, SP – Brazil; 5 Brazil Hospital Sírio Libanês – Instituto de Pesquisa e Ensino, São Paulo, SP – Brazil; 6 Brazil Hospital Israelita Albert Einstein, São Paulo, SP – Brazil; 7 USA Duke University Medical Center, Durham, NC – USA; 8 Brazil Instituto Brasileiro de Pesquisas Clínicas, São Paulo, SP – Brazil

**Keywords:** COVID-19, Thrombosis, Treatment Outcome, Rivaroxaban, Outpatient Clinics, Hospital

## Abstract

**Background:**

Previous studies have demonstrated a high risk of arterial and venous thromboembolic events as a consequence of direct viral damage to endothelial cells by SARS-CoV-2 and a procoagulant milieu due to increased biomarkers, such as D-dimer, fibrinogen, and factor VIII. Although randomized controlled trials of antithrombotic therapies have been conducted in hospitalized patients, few have evaluated the role of thromboprophylaxis in an outpatient setting.

**Objective:**

To assess whether antithrombotic prophylaxis with rivaroxaban reduces the risk of venous or arterial thrombotic events, invasive ventilatory support, and death in COVID-19 outpatients.

**Methods:**

The COVID Antithrombotic Rivaroxaban Evaluation (CARE) study, a multicenter, randomized, open-label, controlled trial of rivaroxaban 10 mg once daily for 14 days or local standard treatment alone to prevent adverse outcomes, is registered in clinicaltrials.gov (NCT04757857). The inclusion criteria are adults with confirmed or suspected SARS-CoV-2 infection and mild or moderate symptoms without indication for hospitalization, within 7 days of symptom onset, and 1 risk factor for COVID-19 complication (> 65 years, hypertension, diabetes mellitus, asthma, chronic obstructive pulmonary disease or other chronic lung diseases, smoking, immunosuppression, or obesity). The primary composite endpoint, which includes venous thromboembolism, invasive mechanical ventilation, major acute cardiovascular events, and mortality within 30 days of randomization, will be assessed according to the intention-to-treat principle. All patients will provide informed consent. A significance level of 5% will be used for all statistical tests.

Clinical trials registration: NCT04757857

**Results:**

Major thrombotic and bleeding outcomes, hospitalizations, and deaths will be centrally adjudicated by an independent clinical events committee blinded to the assigned treatment groups.

**Conclusion:**

The CARE study will provide relevant and contemporary information about the potential role of thromboprophylaxis in outpatients with COVID-19.

## Introduction

The most common symptoms of COVID-19 include fever, fatigue, dry cough, anorexia, myalgia, and dyspnea.^1 - 7^However, patients may progress to severe acute respiratory syndrome (SARS) and multisystem inflammatory syndrome, which involve a high risk of death.^3 - 6 , 8 - 15^Furthermore, there is a high risk of arterial and venous thrombotic events (VTE) from direct viral damage to endothelial cells and increased procoagulant factors, such as D-dimer, fibrinogen, factor VIII. Hyperviscosity has also been reported,^16 - 18^which enhances micro- and macrovascular thrombosis,^19 , 20^in a direct relationship with COVID-19 severity.^21 - 25^Several Brazilian studies in epidemiological and health care contexts have found a series of cardiovascular risk factors associated with both the burden and prognosis of COVID-19.^26 - 29^Randomized controlled trials of antithrombotic therapies in patients with COVID-19 have been conducted in hospitalized patients, especially critically ill patients in intensive care units.^30 - 35^Current guidelines recommend antithrombotic prophylaxis with low molecular weight heparin or unfractionated heparin for hospitalized patients.^36 - 40^However, despite reports of thrombotic events in outpatients,^41^few studies have been published on the frequency and the potential role of thromboprophylaxis.^30^Rivaroxaban is a highly selective, direct inhibitor of factor Xa with proven efficacy against thromboprophylaxis^42 , 43^and secondary cardiovascular events.^44^Selective FXa inhibitors may stop amplified bursts of thrombin production.^45^

The aim of this study is to determine whether rivaroxaban reduces the composite endpoint of VTE, invasive mechanical ventilation, major acute cardiovascular events (defined as acute myocardial infarction, stroke or acute limb ischemia), and death (in or out of the hospital) within 30 days of randomization in COVID-19 outpatients who do not clearly require hospitalization upon initial presentation.

## Methods

### Coalition COVID-19 Brazil

Coalition COVID-19 Brazil is a multicenter research initiative by Brazilian investigators to evaluate the benefits of different drugs on a range of COVID-19 severities. Several studies have been published through this network on the effects of treatments such as azithromycin, hydroxychloroquine, dexamethasone, tocilizumab, and rivaroxaban in moderate to severe patients.^46 - 51^

### Study Design

This multicenter, randomized, open-label, controlled trial with an allocation ratio 1:1 will test whether rivaroxaban (10 mg per os once daily for 14 days) vs. local standard treatment within 7 days of symptom onset will reduce the composite endpoint of VTE, invasive mechanical ventilation, major acute cardiovascular events, and mortality due to COVID-19 within 30 days of randomization. The study will be conducted at 47 sites, based a favorable feasibility assessment, compliance with good clinical practice, and ethics approval. This protocol follows the *Standard Protocol Items: Recommendations for Interventional Trials* statement.^52^The central Illustration shows the study workflow.

**Central Illustration:** : Rivaroxaban in Outpatients with Mild or Moderate COVID-19: Rationale and Design of the Study CARE (CARE – Coalition COVID-19 Brazil VIII)

*CONSORT diagram showing the study workflow and planned recruitment.*

### Primary and Secondary Outcomes

We will assess the composite efficacy endpoint of VTE, invasive mechanical ventilation, major acute cardiovascular events, and 30-day mortality (in or out of the hospital). We also plan to evaluate the time from randomization to hospitalization, length of hospital stay, admission to the intensive care unit, clinical requirements, duration of mechanical ventilation, composite vascular endpoint I (ie, non-fatal myocardial infarction, non-fatal ischemic stroke, cardiovascular death, or VTE), composite vascular endpoint II (ie, cardiovascular death, non-fatal myocardial infarction, non-fatal ischemic stroke, acute limb ischemia, or VTE), major bleeding, defined according to International Society on Thrombosis and Hemostasis criteria:^53^a) any sign or symptom of hemorrhage (more bleeding than would be expected for a clinical circumstance, including bleeding found by imaging alone) that does not fit the criteria for major bleeding but meets at least one of the following: i) requiring medical intervention; ii) leading to hospitalization or a higher level of care; iii) prompting a face-to-face evaluation (not by telephone or electronic means); b) major bleeding in non-surgical patients, defined as having a symptomatic presentation, and: i) fatal bleeding, and/or ii) bleeding in a critical area or organ, eg, intracranial, intraspinal, intraocular, retroperitoneal, intra-articular or pericardial, or intramuscular with compartment syndrome, and/or iii) bleeding that causes a ≥ 20 g.L^1^(1.24 mmol.L^1^) decrease in hemoglobin level or that leads to a transfusion of ≥ 2 units of whole blood or red cells; in addition to all-cause mortality.

### Eligibility Criteria

Suspected and confirmed COVID-19 cases will be initially assessed based on Brazilian Ministry of Health guidelines and WHO recommendations^54 , 55^and modified to the outpatient setting ( Table 1 ). The inclusion and exclusion criteria are described in Table 2 .

**Table 1:** – COVID-19 case definition for the CARE Study according to Brazilian Ministry of Health and World Health Organization criteria

COVID-19 status	Definition
Confirmed	Patients with a laboratory confirmed COVID-19 diagnosis by RT-PCR, preferably collected between the fourth and seventh days after symptom onset by nasopharyngeal/oropharyngeal swab, regardless of signs and symptoms. Validated rapid immunochromatographic assay for viral antigen detection in sample collected from the upper respiratory tract, preferably between the second and seventh days after sign/symptom onset. Immunological (rapid test or classic serology to detect IgM/IgG antibodies), in a sample collected on at least the seventh day after symptom onset, analyzed by a validated test.
Suspected	Patients who meet at least one of the following criteria *: Patients with acute respiratory disease (fever AND at least 1 sign/symptom of respiratory disease, eg, cough or dyspnea), AND a history of travel or residence in a location that has reported the community transmission of COVID-19 during the 14 days prior to symptom onset. Patient with acute respiratory disease AND contact with a confirmed or probable case of COVID-19 (acute respiratory disease without laboratory confirmation) in the 14 days prior to symptom onset.

* Depending on the patient’s clinical status, these criteria can be complemented by radiological findings (interstitial infiltration in a chest X-ray and/or ground-glass opacity in a lung computed tomography scan). It should be noted that the majority of participants will have mild symptoms and will not be clinically indicated for imaging tests. RT-PCR: real-time polymerase chain reaction.

**Table 2:** – Inclusion and exclusion criteria

General Criteria
Adult outpatients (aged > 18 years) who seek medical care and have a suspected or confirmed diagnosis of COVID-19, are within 7 days of symptom onset, present with mild to moderate symptoms, have no clear indication for hospitalization, and at least two of the following risk factors for complications:

**Inclusion Criteria**

Age ≥ 65 years Hypertension Diabetes mellitus Asthma COPD or other chronic lung diseases Smoking Immunosuppression Obesity (BMI > 30) History of non-active cancer Bed restriction or reduced mobility (≥ 50% of waking time without walking)) Previous history of venous thromboembolic events Use of oral hormonal contraceptives

**Exclusion Criteria**

Patients aged <18 years Hospitalization indicated at initial treatment Positive test for influenza at the first visit Any known liver disease associated with coagulopathy; INR > 1.5 Women who are pregnant, lactating, may become pregnant, or are not using an adequate contraception method Patients with a high risk of bleeding, a history of bronchiectasis or pulmonary cavitation, significant bleeding in the last 3 months, an active gastroduodenal ulcer, a history of recent bleeding (within 3 months), or a high risk of bleeding Stroke in the last month, a history of hemorrhagic or lacunar stroke, any intracranial bleeding or neoplasia, brain metastasis, arteriovenous malformation, or brain aneurysm Severe heart failure with left ventricular ejection fraction < 30% (identified by echocardiogram or another previously validated method) or symptoms of New York Heart Association heart failure class III or IV Estimated glomerular filtration rate < 30 mL/min according to the CKD-EPI equation Clinical indication for dual antiplatelet therapy or anticoagulation therapy (venous thromboembolic events, atrial fibrillation/flutter, or mechanical valve prosthesis) Marked thrombocytopenia (platelets < 50,000/mm3) Non-cardiovascular disease associated with a poor prognosis, eg, active cancer (excluding non-melanoma skin cancer), defined as cancer not in remission or that requires active chemotherapy or adjuvant therapies, such as immunotherapy or radiation therapy, or that increases the risk of an adverse reaction to the interventions A history of hypersensitivity or known contraindication to rivaroxaban Systemic treatment with strong inhibitors of CYP3A4 and P-glycoprotein (systemic azole antimycotics, such as ketoconazole, or HIV protein inhibitors, such as ritonavir) or strong inducers of CYP3A4, ie, rifampicin, rifabutin, phenobarbital, phenytoin, and carbamazepine Current treatment in test phase Concomitant participation in another COVID-19 study involving experimental drugs Use of chloroquine or hydroxychloroquine associated with azithromycin Active cancer Other contraindications to rivaroxaban

*CKD-EPI: Chronic kidney disease epidemiology collaboration; INR: international normalized ratio.*

### Trial Interventions and Follow-up Assessment

Both arms will receive local standard treatment, which includes general advice and medications for symptom relief (left to the physician’s discretion). Patients in the rivaroxaban group will receive 10 mg once daily per oral administration for 14 days. Patients in the control group are not expected to receive additional treatment beyond local care, as previously mentioned. Two follow-up visits are planned via telephone to evaluate adherence to rivaroxaban and to detect disease progression or adverse events.

### Discontinuation of the study medication and patient follow-up

The investigator will discontinue the study drug and the patient should be followed up for 30 days, with all of the following procedures carried out:

- any medical condition that, at the sponsor’s or investigator’s discretion, prevents the patient from continuing treatment, describing the reason and providing proof thereof;
- patients who develop a serious adverse event that compromises adherence and safety. The investigator will follow up such patients until the adverse event is resolved or stabilized and considered clinically irrelevant.
- Patients with negative SARS-CoV-2 results in a real-time polymerase chain reaction.

When the SARS-Cov-2 test is performed at the hospital where the patient is randomized, the center’s principal investigator will obtain the results and send them to the coordinating center (International Research Center at the Hospital Alemão Oswaldo Cruz); the patient will be asked to discontinue treatment and will be followed-up until the end of the 30-day period.

When the real-time polymerase chain reaction test for SARS-Cov-2 is performed at another laboratory, patients will inform the center to which they are randomized. This center will then inform the International Research Center so that the study team can indicate interrupting the study drug, with follow-up continuing for up to 30 days.

The study will be interrupted if the intervention shows clear benefits at the primary efficacy endpoint or if the frequency of major bleeding events increases. The Data and Safety Monitoring Board will closely monitor any serious adverse events and, using statistical assumptions, could recommend ending the study to ensure patient safety.

### Adverse Event Reporting and Management

The retrieved information should include the medical history and comorbidities, and clinical diagnosis of COVID-19, in addition to its severity, start date, main cause and the likelihood of a causal relationship, medical decisions, adverse outcomes, the criteria used to classify the severity of the event, and the end date.

### Endpoint Reporting and Adjudication

The primary efficacy endpoint will be evaluated by physicians with longstanding experience in clinical event adjudication. Hospital admissions for causes related to COVID-19 will be documented by the medical team, with the information collected for clinical analysis under blinded allocation according to standardized criteria similar to those used in the Prospective Urban and Rural Epidemiology study.

### Data Collection and Management

Data will be collected using an online case report form and entered at each participating site. Training and support will be provided by the coordinating center to ensure data quality. Data to be collected from the patient and/or family during study visits include:

- 1. Admission (Baseline)

- Age, sex, marital status, race, education, family income, and comorbidities
- The results of molecular or serological tests for COVID-19 (depending on the time since symptom onset/clinical diagnosis)
- Concomitant medications at baseline
- Symptom duration

- 2. On the 15th day post-randomization

- Safety assessment (Adverse event monitoring)
- Medication adherence

- 3. On the 30th day post-randomization

- Efficacy assessment (primary outcome events)
- Safety assessment (adverse event monitoring)

The data collection and participant follow-up plans are shown in Figure 1 .

**Figure 1:** – Data collection and patient follow-up plan.

### Statistical Analysis

#### Sample size calculation and randomization procedure

We estimated that a sample size of 932 patients (466 per group) would provide 80% statistical power to detect a relative risk reduction of 30%, assuming a primary endpoint rate of 25% in the control group and 17.5% in the rivaroxaban group, a two-sided hypothesis test, and 5% significance level using the chi-square test. With an estimated follow-up loss of 5% per group, the total would increase 982 patients, therefore 1000 patients will be randomized. The sample size was calculated using SAS 9.4 (PROC POWER procedure).^56 , 57^A permuted-block randomization technique will be applied with fixed block size of 8 through a randomization list produced electronically using appropriate software.^58 , 59^Randomization concealment will be maintained through a 24-hour, centralized, automated, Internet-based system developed by the Hospital Alemão Oswaldo Cruz.

#### Interim analyses

Three interim analyses are planned by an independent Data and Safety Monitoring Board when the sample size reaches 25% (250 individuals), 50% (500 individuals), and 75% (750 individuals), according to Haybittle–Peto analysis.^60 , 61^After the safety analysis, the study may be interrupted if there is a sign of harm with p < 0.01 (in each interim analysis). In the efficacy analysis, the study may be interrupted if there is a sign of benefit (primary outcome) with p < 0.001 (in each interim analysis).^62^

#### Statistical Methods

Baseline characteristics will be reported as counts, percentages, and means and standard deviations or medians and interquartile ranges, when appropriate.^63^The effect of rivaroxaban on the primary and secondary endpoints will be estimated using risk ratios and 95% confidence intervals. All endpoints will be assessed in the intent-to-treat population. The chi-square test or Fisher’s exact test will be used for hypothesis testing. Absolute differences between two proportions with 95% CI will be also estimated according to Newcombe’s method.^64 , 65^Exploratory analyses for the primary outcome will be performed considering the intervention effect in prespecified or post-hoc subgroups, according to observations throughout the study, and dynamic changes in SARS-CoV-2 variants. Interaction tests will be conducted using binary logistic regression models that include the treatment effect, the factor of interest, and an interaction term between the 2 variables (treatment-by-subgroup interaction term) using the full patient set, reporting the p-value for the interaction term.^66^The effect of the intervention on mortality will be assessed using Kaplan-Meier curves and univariate Cox proportional hazard regression modeling, with hazard ratios and 95% CI. Survival curves will be compared using log-rank tests. Proportional hazard assumptions will be checked using cumulative sums of martingale residuals and a Kolmogorov-type supremum test based on a sample of 1000 simulated residual patterns.^67 , 68^If the phenomenon of monotone likelihood occurs or few events are observed, Firth’s penalized partial likelihood approach will be applied in a Cox regression model, with hazard ratios and 95% profile-likelihood confidence limits.^69 , 70^Based on patient recruitment and epidemiological data (continuously updated by the Ministry of Health and local reports), we plan to include three additional methods that might contribute to the overall clinical efficacy assessment: a) the primary efficacy endpoint plus hospitalizations due to COVID-19, b) the win ratio for the primary efficacy endpoint, and c) the win ratio for the primary efficacy endpoint plus hospitalizations due to COVID-19.^71 , 72^Secondary outcomes, defined by quantitative variables, will be compared between the groups using an unpaired Student’s *t* -test or the Mann-Whitney test for non-normally distributed variables, when appropriate. Adverse events will be compared between the treatment groups using the chi-square test or Fisher’s exact test. Additionally, if the proportion of missing data exceeds 5% for the primary outcome,^73 , 74^a sensitivity analysis will be conducted using a multiple data imputation technique.^75 , 76^Normality will be assessed through visual inspection of histograms and the application of normality tests, such as the Shapiro-Wilk or D’Agostino-Pearson test, when appropriate.^77 , 78^All hypothesis tests will be two-sided, with p < 0.05 considered statistically significant. Statistical analyses will be conducted using SAS version 9.4 (SAS Institute Inc., Cary, NC, USA) or R Statistical Software (R Foundation, Vienna, Austria).

#### Database lock

Database lock will occur after completing the 30-day follow-up of all patients, final clinical monitoring, and data cleaning. All interim analyses will be made available to local regulatory agencies in Brazil. Database access will be granted only to the Steering Committee members and statisticians before the main results are published.

#### Trial oversight

An Executive/Steering Committee will be responsible for general supervision of the study, developing the study protocol, and writing the manuscript. The Data and Safety Monitoring Board will assess the effects of rivaroxaban compared to local standard treatment in relation to the primary and secondary endpoints. This committee will monitor serious adverse events and may recommend treatment interruption, if necessary, to ensure patient safety.

## Ethics and dissemination

The records of all patients will be kept confidential and with restricted access. Each patient or legal representative will sign an informed consent form after the study’s risks/benefits and procedures have been fully explained. The initial protocol and all amendments have been approved by local institutional review boards and the Brazilian National Ethics Research Commission, in compliance with the ethics Resolution CNS 466/2012. This trial has been registered at clinicaltrials.gov (NCT04757857) and will be submitted for publication, regardless of its results.

## Perspective

The CARE study will provide relevant and contemporary information about the potential role of thromboprophylaxis in outpatients with COVID-19.

## Funding sources

This investigator-initiated study has received financial support from the COALITION COVID-19 Brazil and Bayer S.A., who provided the study drug and partial financial support. The funding sources had no role in the study design, data analysis and interpretation, or the decision to publish the results.

## Data Sharing Statement

All information regarding this clinical trial protocol has been provided in this publication.
